# Therapeutic Potential of Hispidin—Fungal and Plant Polyketide

**DOI:** 10.3390/jof7050323

**Published:** 2021-04-22

**Authors:** Kseniia A. Palkina, Daria A. Ipatova, Ekaterina S. Shakhova, Anastasia V. Balakireva, Nadezhda M. Markina

**Affiliations:** 1 Department of Biomolecular Chemistry, Shemyakin-Ovchinnikov Institute of Bioorganic Chemistry of the Russian Academy of Sciences, 117997 Moscow, Russia; ksenia.palkina@planta.bio (K.A.P.); ipatova.daria@yandex.ru (D.A.I.); ekaterina.shakhova@planta.bio (E.S.S.); anastasia@planta.bio (A.V.B.); 2 Planta LLC, 121205 Moscow, Russia; 3 School of Pharmacy, Faculty of Biomedicine, Pirogov Russian National Research Medical University, 117997 Moscow, Russia

**Keywords:** hispidin, polyketides, antioxidant, therapeutic potential

## Abstract

There is a large number of bioactive polyketides well-known for their anticancer, antibiotic, cholesterol-lowering, and other therapeutic functions, and hispidin is among them. It is a highly abundant secondary plant and fungal metabolite, which is investigated in research devoted to cancer, metabolic syndrome, cardiovascular, neurodegenerative, and viral diseases. This review summarizes over 20 years of hispidin studies of its antioxidant, anti-inflammatory, anti-apoptotic, antiviral, and anti-cancer cell activity.

## 1. Introduction

People have been using fungi as nutrients and the source of natural bioactive compounds since ancient times. Fungi have become a part of folk and traditional medicine due to the unique medicinal properties of some species [1,2]. Thus, the extracts of mushrooms that belong to the *Phellinus* and *Inonotus* genera have been used for the treatment of various diseases including malignant tumors, cardiovascular and liver diseases, diabetes, and others [3,4]. The exact mechanisms underlying the medicinal activity of fungal extracts are still mostly unknown. However, the main role of treating agents was attributed to the number of polysaccharides [3,4,5] and, more recently, to the low-molecular-weight bioactive compounds. Among the latter, there are styrylpyrones—polyphenol pigments that display a large number of biological effects. One of the first discovered styrylpyrones was polyketide hispidin isolated from *Inonotus hispidus* fungi in 1889, its structure was identified in 1961 [5,6]. Hispidin was found in various related fungi species of the *Hymenochaetaceae* family (genus *Inonotus (I. obliquus, I. xeranticus), Phellinus* (*P. linteus* also known as *Sanghuangporus sanghuang* [7], *P. ignarius*, *P. baumii*, *P. harmala*, *P. sensulato*)) and the Australian fungus *Cortinarius* [8]. Hispidin was also identified in luminescent mushrooms *Neonothopanus nambi* as a key precursor of fungal luciferin [9]. Moreover, hispidin and its derivatives were discovered in many plants such as the horsetail (*Equisetum arvense)* [10], piper (*Piper methysticum)* [11], the *Compositae* plant achirocline (*Achyrocline bogotensis)* [12], pistachio (*Pistacia atlantica)* [13], and others.

**Figure 1:**
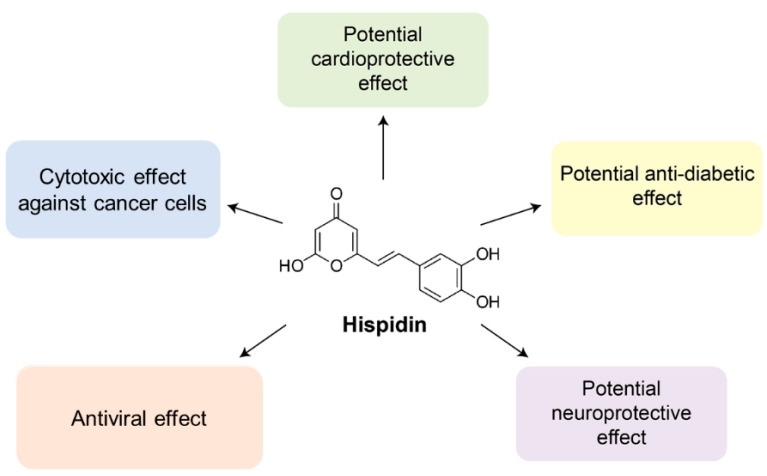
Hispidin exhibits cytotoxic, anti-diabetic, anti-inflammatory, antiviral, cardioprotective, and neuroprotective effects covered in the present review.

To date, hispidin is one of the best-studied polyketides among other fungal polyphenols. It can neutralize free radicals [3,14,15,16,17]. Recent studies have shown that hispidin but not polysaccharides or flavonols determine the antioxidant and antitumor properties of *Phenllinus* mushroom extracts [18]. Besides antioxidant activity hispidin displays cytotoxic [19], potentially hypoglycemic [20], anti-inflammatory [21], antiviral [22], and neuroprotective properties [23]. At the same time, the Ames test, in vitro chromosome aberration test, acute oral toxicity test, and bone marrow micronucleus test demonstrate a very low toxicity for human consumptions [7]. As hispidin is a promising bioactive compound for the potential application in biomedicine, herein we summarize the available data on the effects of hispidin (Figure 1).

## 2. Cytotoxic Effect of Hispidin on Cancer Cells

The World Health Organization represents cancer as one of the main death causes of the past and ongoing centuries [24]. As a universal approach to cancer treatment is absent, the search for novel compounds exhibiting anticancer activities is still relevant [25]. Natural compounds are particularly attractive due to the virtual lack of side effects compared to synthetic analogs [26]. Secondary metabolites of plants and fungi, including polyketides, can serve as a bioactive compound source [27].

Gonindard and colleagues in 1997 have shown the hispidin-induced in vitro cytotoxic effects on two different cancer cell lines including skin squamous cell carcinoma SCL-1 and pancreatic ductal adenocarcinoma Capan-1 [19]. Later a similar cytotoxic effect of hispidin was observed for other cancer cell lines. The list of cell lines susceptible to hispidin can be found in Table 1. According to published data cytotoxic effect of hispidin is achieved by activating various mechanisms, which will be considered below.

One of the mechanisms is related to protein kinase C (PKC), which isoforms are among the key participants of carcinogenesis. Despite under normal circumstances PKC is responsible for cell homeostasis maintenance, an increase in the expression level of PKC isoforms is often associated with malignant tumor growth [32]. The cytotoxic effect of hispidin is presumably achieved via inhibition of the activity of PKC isoform β through an unclear exact mechanism [19,33]. This ability of hispidin to inhibit PKC activity was successfully applied in the studies of PKC activation-dependent cell processes [34,35].

The alternative mechanism of cytotoxic effect of hispidin is described for the human papillomavirus-related endocervical adenocarcinoma SGC-7901 cell line. According to Lv and co-authors, this polyketide induces autophagic and necrotic death of adenocarcinoma cells but does not show the cytotoxic effect on control cells such as human liver cell line L02 and stomach cell line GES-1 [30]. Hispidin activates phosphorylation of stathmin-1 at Ser16 which causes depolymerization of microtubules in SGC-7901. Cancer cell microtubules oscillate and increase the membrane permeability of peripheral lysosomes, which become large and fragile compared to the normal ones [36]. This mechanism determines the cytotoxic effect of hispidin on SGC-7901, human lung adenocarcinoma A549 and human hepatocellular carcinoma HepG2 [30].

**Figure 2:**
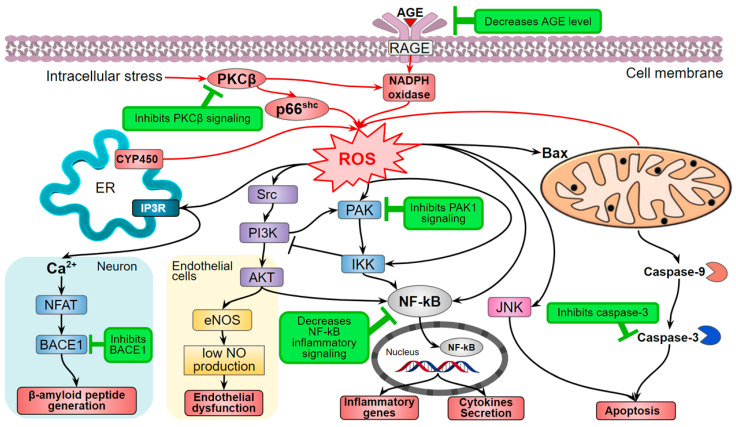
The hispidin effect on ROS production and ROS-dependent signaling (marked green). AGE—Advanced glycation end-product; AKT—Protein kinase B; BACE1—β-site amyloid precursor protein cleaving enzyme 1 (β-secretase); Bax—Bcl-2-associated X protein; CYP450—cytochrome P450; eNOS—Endothelial nitric oxide synthase; ER—Endoplasmic reticulum; IKK—IκB kinase; JNK—Jun N-Terminal protein kinase; NF-kB—Nuclear factor-kappa B; NFAT—Nuclear factor of activated T-cells; p66^Shc^—isoform of SHC1 adaptor protein; PAK—Serine/threonine-protein kinase; PKCβ—protein kinase C β-isoform; RAGE—Receptor for AGE; ROS—reactive oxygen species; Src—Proto-oncogene tyrosine-protein kinase.

The Shinkichi Tawata research group suggested the mechanism of indirect influence of hispidin and a number of other natural compounds through inhibtion of serine/threonine-protein kinase 1 (PAK1)-dependent signaling. This assumption was corroborated with the experiments on mouse B16F10 melanoma and human A549 lung adenocarcinoma cell lines [29,37]. Hispidin decreases ROS production and ROS-dependent signaling, which is consistent with other studies (Figure 2).

The antioxidant properties of hispidin are noted in many studies [38,39,40,41]. Despite this fact, the study by Lim et al. describes that hispidin-induced ROS-dependent apoptosis in CMT-93 mouse colon cancer cells and HCT 116 human colon cancer cells [28]. According to the experimental results, hispidin increases ROS level in a dose-dependent manner. In addition, hispidin-dependent apoptosis manifests in chromatin condensation, nuclear fragmentation, and the cells acquiring an abnormal spherical shape. It is worth noting that a similar effect was never described by other research groups. In this regard, it is possible that the ROS level increase is hispidin-independent, and the exact mechanism of apoptosis, in this case, remains unknown.

Another mechanism of impact of hispidin on cancer cell viability comprises influence on the key transcription factor Nuclear factor-kappa B (NF-kB), which is crucial for cycle maintenance, stress adaptation, inflammation, and apoptosis. The malfunctioning of NF-kB can cause oncogenesis, malignant tumor invasion, autoimmune disorders, metastasis, and resistance to antitumor therapy [42,43]. Chandimali and colleagues have shown that hispidin induced apoptosis and inhibited proliferation of pancreatic ductal adenocarcinoma BxPC-3 and AsPC1 cells. Hispidin inhibited NF-kB and enhanced the activity of p53 (known as a malignant tumor suppressor), caspase-3, and poly-ADP-ribose polymerase expression [31]. Thus, hispidin shows cytotoxic effects on cancer cells.

Hispidin increased sister chromatid exchange frequency and decreased replication index and nuclear division index values in human lymphocytes in vitro without other DNA damage [44]. Perhaps, the same mechanism partially underlies the cytotoxic effect of hispidin on cancer cells.

Furthermore, hispidin enhances the biological activities of other compounds such as chemotherapy drug gemcitabine: hispidin sensitized pancreatic cancer stem cells to gemcitabine and promoted its therapeutic efficacy [31,45].

## 3. The Effect of Hispidin on the Metabolic Syndrome

Metabolic syndrome is a worldwide condition comprising carbohydrate metabolism disorders, abdominal obesity, hypertension, and dyslipidemia that characterize diabetes mellitus and accompanying cardiovascular complications [46]. This disorder is strongly associated with pathological processes like oxidative stress and inflammation [47]. Therefore, scientists struggle to discover new natural compounds and to derive novel drugs applicable for the treatment and prevention of obesity, insulin resistance, and other accompanying complications.

**Figure 3:**
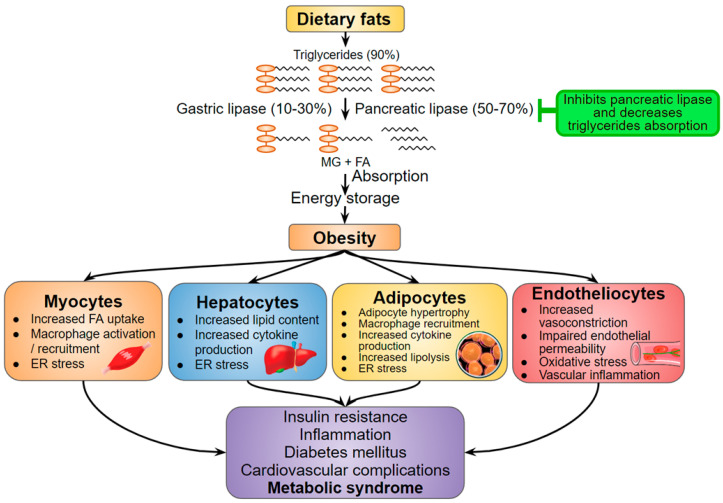
Hispidin as a potential anti-obesity compound (marked green) in the context of metabolic syndrome developing. Adapted from [50,53]. MG—Monoacylglycerol; FA—Fatty acids; ER stress—Endoplasmic reticulum stress.

Abdominal obesity and increased free fatty acids lead to inflammation and oxidative stress that provoke insulin resistance and β-cells dysfunction [48]. To avoid ectopic fat accumulation and, therefore, prevent obesity, the treatment should stimulate lipolysis and inhibit the absorption of food triglycerides [49,50]. Hispidin in vitro inactivates pancreatic lipase — an important anti-inflammatory target (Figure 3) that decreases triglyceride absorption in the small intestine. In addition, hispidin inhibits glycerol-3-phosphate dehydrogenase, which serves as an important link between carbohydrate and lipid metabolism, and, as a consequence, decreases intracellular triglyceride levels in a dose-dependent manner [51]. Hispidin is also able to increase the intracellular cAMP level [51], which is supposedly associated with enhanced lipolytic signaling [52]. Concluding, hispidin can suppress lipohypertrophy and triglycerides absorption.

**Figure 4:**
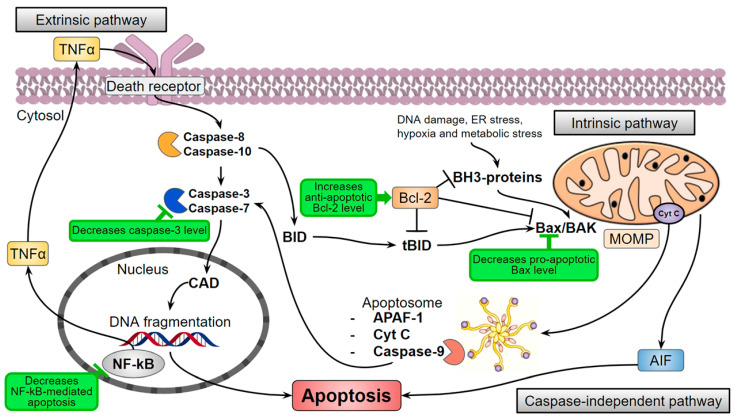
Hispidin (marked green) can influence the levels of apoptotic pathways participants. AIF—Apoptosis-inducing factor; APAF-1—Apoptotic protease activating factor-1; BAK—Bcl-2 homologous antagonist/killer; Bax—Bcl-2-associated X protein, pro-apoptotic regulator; Bcl-2—Anti-apoptotic regulator protein; BID—Pro-apoptotic protein of the Bcl-2 family; CAD—Caspase-activated DNase; Cyt C—Cytochrome C; ER stress—Endoplasmic reticulum stress; MOMP—Mitochondrial outer membrane permeabilization; NF-kB—Nuclear factor-kappa B; TNFα—Tumor necrosis factor-alpha.

Triglycerides accumulation causes elevated oxidative stress that can lead to the death of ROS-sensitive cells. It is especially important for skeletal muscles that utilize about 75% of available in the organism glucose in their normal postprandial state [54] and are very sensitive to lipotoxicity, inflammation, and oxidation [55]. For instance, the palmitate-induced obesity model of immortalized mouse myoblasts (myotubes C2C12) provoked cell death but hispidin increased the cell survival through the inhibition of the key effector protease in apoptosis-caspase-3 [38] (Figure 4). In addition, the pro-apoptotic protein Bax level is decreased, and the anti-apoptotic protein Bcl-2 level is increased. Moreover, hispidin inhibited the translocation of the nuclear factor NF-kB—the major inflammation regulator—and prevented cell apoptosis through the NF-kB signaling pathway in the hyperglycemic model of rat pheochromocytoma PC12 cells [40]. The antioxidant effect of hispidin contributes to the leveling of oxidative stress caused by ROS, but the mechanism remains unknown.

Elevated oxidative stress and inflammation also induce insulin resistance: the inability of insulin-dependent cells to respond to insulin stimulation, which normally enhances the glucose uptake from the blood. Thus, insulin resistance results in excessive glucose accumulation in the blood—hyperglycemia, a key component of diabetes mellitus pathogenesis. Hyperglycemia leads to even more elevated oxidative and carbonyl stress and further progression of metabolic disorders. The increased ROS concentration follows an increased level of advanced glycation end-product (AGE), activation of AGE receptor (RAGE), PKC activity, and the hexosamine and polyol metabolic pathways [56,57]. Enhanced non-enzymatic protein glycation is one of the major hyperglycemia problems that provokes the activation of RAGE, NF-kB pro-inflammation signaling, and increases oxidative stress (Figure 2) [58]. In this context, hispidin and its fungal derivatives demonstrate both direct free radical activity and antiglycation effect that not only reduces the oxidative stress but also compensates the damage caused by AGE and ROS [59]. In addition, hispidin is better than unnatural AGE inhibitors that exhibit poor pharmacokinetics [60].

The increase in glucose levels also stimulates its consumption through the polyol pathway—two-step conversion of glucose to fructose. High activity of its key enzyme—aldose reductase—leads to the depletion of the cellular NADPH pool, which shifts the oxidative-antioxidant balance and elevates oxidative stress [57]. Hispidin inhibits aldose reductase, which is a potential therapeutic target for the prevention of hyperglycemia-associated complications [20].

The treatment of diabetes mellitus and insulin resistance also targets tyrosine phosphatase 1β (PTP1β) [61] which blocks insulin-dependent signaling by insulin receptor dephosphorylation [62]. Presumably, PTP1β inhibitors increase insulin receptor sensitivity, recover its autophosphorylation and activate the downstream signaling cascades [63]. A number of low-molecular-weight PTP1β inhibitors have been described including ones undergone clinical trials [64] but the search for more effective pharmaceuticals is still ongoing. Thus, the ability of hispidin isolated from *Phellinus linteus* to inhibit PTP1β [65] arouses great interest in further study of mechanisms of hispidin interaction with the target.

Decreased sensitivity of muscle, fat, and liver cells to insulin stimulation forces pancreatic β-cells to synthesize more insulin that inevitably leads to their depletion. This pathologic process is enhanced by developing inflammation and oxidative stress that causes the apoptosis of β-cells. Therefore, the dysfunction and death of pancreatic β-cells are other important factors in pathophysiology of diabetes mellitus [66]. Hispidin showed the protective effect in the model of pancreatic β-cells RINm5F treated with hydrogen peroxide. H_2_O_2_ induces oxidative stress, which, in turn, suppresses insulin production by pancreatic β-cells in the RINm5F and MIN6N lines. However, hispidin-reduced cell apoptosis and also promoted the maintenance of insulin secretion even in the presence of hydrogen peroxide [39,41]. The hispidin-dependent reduction of free radicals and intracellular ROS levels are comparable to other antioxidants such as N-acetyl-L-cysteine and vitamin C [39]. Besides, unlike synthetic antioxidants such as butylated hydroxyanisole and hydroxytoluene, hispidin is not toxic and therefore is a prospective insulin production-associated protective agent [67].

The mentioned protective effects of hispidin should provoke a further molecular mechanisms comprehensive study of this influence on inflammation processes, oxidative and carbonyl stress, as well as lipid metabolism. Potentially, it would be effective for the development of metabolic syndrome therapy methods.

## 4. Hispidin as a Potential Cardiovascular Protector

Cardiovascular diseases can evolve as an independent pathological process or as complications of metabolic disturbance such as diabetes mellitus. Oxidative stress is an important risk factor for the development of cardiovascular diseases [68]. ROS provoke apoptosis in the cardiomyocytes and, as a consequence, lead to ischemia and myocardial infarction [69]. They also cause and enhance endothelial dysfunction that leads to vascular damage and the development of atherosclerosis [70].

Hispidin demonstrated the antioxidant effect on H9c2 cardiomyoblast cells treated with hydrogen peroxide. Hispidin stimulated a decrease of intracellular ROS, an increase of antioxidant enzymes (heme oxygenase-1 and catalase), and activation of Akt, GSK-3β, and ERK1/2, and whole phosphatidylinositol-3-kinase signaling pathway in H9c2 cells [71]. These kinases control the metabolism and death of the cells, so they could be considered as potential therapeutic targets for the treatment of cardiovascular diseases [72].

Restoration of anti-apoptotic Bcl-2 and pro-apoptotic Bax proteins ratio suppresses the death of cardiomyoblasts H9c2 and likely increases cell survival under the influence of hispidin [73,74]. Hispidin also inhibits caspase-3 in H9c2 in a dose-dependent manner that increases the survival of the cells (Figure 4) [71].

During hyperlipidemia, free fatty acids accumulate in cells and lead to the formation of diacylglycerols—activators of PKC. Stimulation of PKC signaling in blood vessels provokes the development of insulin resistance, impaired NO production, and the development of endothelial dysfunction [75]. A number of studies rely on the hispidin ability to specifically inhibit the β isoform of PKC, including endothelial cell models HUVEC and HCAEC [76,77]. Another research group analyzed hispidin influence on the endothelial barrier function regulated by PKC signaling [78]. Unfortunately, no one has yet studied the effect of this polyketide on the functioning of vascular cells, NO production, and intracellular signal transmission.

Cancer patients are prone to the risk of cardiovascular diseases [79]. Antitumor therapy can lead to ischemia and oxidative stress-induced reperfusion development [80]. Therefore, the authors investigated the effect of hispidin in the doxorubicin-induced cytotoxic model cardiomyoblast cells H9c2 and showed that hispidin suppresses the apoptotic activation of caspase-9 (Figure 4) [81]. In addition, PKCβ isoform activates adapter protein and oxidative stress sensor—p66^Shc^—that leads to the formation of ROS [82] (Figure 2). Sampaio and coauthors showed that hispidin inhibits the subcellular movement of p66^Shc^ thus decreasing oxidative stress. Thus, the protective effect of hispidin is based on its antioxidant PKS inhibition activity [81]. Nevertheless, this is not always sufficient to suppress the cytotoxic influence of the oncosuppressors, presumably due to a caspase-independent pathway of cell death [83]. Hispidin also inhibited phosphorylation of p66^Shc^ Ser36 in fibroblasts of Leigh syndrome patients, which led to decreased ROS production and intracellular oxidative stress [84].

Thereby, the cardioprotective effect of hispidin is mainly associated with its antioxidant properties and the ability to specifically inhibit PKC activity as well as to suppress cardiomyoblasts apoptosis by various mechanisms.

## 5. Potential Neuroprotective Effect of Hispidin

Neurodegenerative diseases are a group of disorders with complex dysfunction of the nervous system and sensory organs. Commonly, these pathological processes are associated with metabolic disorders, deficiency of several compounds (thiamine, B12), or exogenous toxins effects [85]. They can also occur as a result of mitochondrial dysfunctions and oxidative stress caused by ROS [86,87].

Age is a risk factor for neurodegeneration accompanied by increased oxidative stress. Thus, high ROS levels provoke retinal pigment epithelial cells macular degeneration—leading cause of progressive blindness [88]. Hispidin incubated with adult retinal pigment epithelial ARPE-19 cells enhanced the expression of nuclear factor erythroid 2-related factor 2 (Nrf2) and its target genes, coding antioxidant enzymes (HO-1, NQO-1, GCLM, and GCLC) [89]. These results demonstrate hispidin antioxidative activity by activation of the JNK-pathway and Nrf2 signaling.

Oxidative stress impairs mitochondrial metabolism. Patients with such disorders suffer from multiple metabolic dysfunctions such as mitochondrial encephalopathy, deafness, retinitis pigmentosa, etc. Hispidin exhibits antioxidant activity and acts as an active substance in antioxidant defense reactions in fibroblasts, derived from patients with mitochondrial disorders. Long-term incubation of fibroblast cells with hispidin did not change mitochondrial bioenergetic parameters significantly, but the production of cytosolic superoxide decreased [90].

Oxidative stress also causes Parkinson’s disease, though its pathogenesis is poorly studied [91]. Hispidin activity in suppression of p66^Shc^ 6-hydroxydopamine-induced phosphorylation reduces hydroxydopamine cytotoxicity [92]. Inhibition of β-site amyloid precursor protein cleaving enzyme 1 (BACE1) is one of the ways of dementia and Alzheimer’s disease treatment. BACE1 stimulates the release of a toxic β-amyloid peptide in the brain [93]. Hispidin inhibits BACE1 and as a consequence decreases the accumulation of β-amyloid peptide [94] (Figure 2).

Nitrative stress also provokes pathogenic mechanisms that lead to neuronal degeneration. A high concentration of peroxynitrite mediates this stress and causes neuron degeneration [95]. Oxidation induces such a dangerous initial injury as DNA breaks, which, furthermore, cause necrosis and apoptosis of neurons [96]. Hispidin is reported to reduce cytotoxicity and inhibit peroxynitrite-induced DNA damage [97]. Shin and colleagues recommend using hispidin for cell protection against methamphetamine-induced neurotoxicity [98].

## 6. Antiviral Effects of Hispidin

The medicinal properties of fungi have been described a long time ago, however, the antiviral effect of hispidin is poorly studied and is mentioned only in several works. Neuraminidase is one of the first described targets that hispidin inhibits. This viral enzyme is responsible for the hydrolysis of terminal neuraminic acid residues of virions and host cell receptors that lead to the release of virions from the infected cell. Hispidin obtained from the extract of *P. baumii* fruit bodies demonstrated inhibitory activity to H1N1, H5N1, and H3N2 neuraminidases at the same level as commonly used neuraminidase inhibitor zanamivir [99]. Inhibition of neuraminidases was also shown using hispidin extracted from *I. hispidus* fruit body [22] and *P. linteus* culture [100].

Hispidin was predicted as an inhibitor of the SARS-Cov2 main protease. Serseg and co-authors showed that hispidin forms highly affinable hydrogen bonds with 2019-nCoV protease residues. This group also recommended using hispidin in medical practice and supposed that hispidin could become a candidate for antiCOVID-19 drug [101,102].

## 7. Conclusions

Free radicals are involved in the pathogenesis of cardiovascular, neurodegenerative, metabolic diseases, as well as in cancer initiation and progression [103]. Many studies highlight that hispidin scavenges free radicals that raises the interest for further study of the mechanisms of this phenomenon as well as its medicinal properties (Table 2). Experimental and theoretical studies of the correlation between the structure of polyphenols and their antioxidant activity have shown that it mainly depends on phenolic hydroxyl groups and their ability to donate a hydrogen atom to quench free radicals [104,105]. Thermodynamic and kinetic studies have shown that the proton-coupled electron transfer is the main mechanism for getting rid of free radicals by hispidin and its analogs, which were found in the *Hymenochaetaceae* family of fungi [16]. The anti-diabetic, cardioprotective, and cytoprotective properties of hispidin are likely caused by the reduction of oxidative damage and activation of signaling cascades that stimulate the internal protective resources of cells.

The other crucial activity of hispidin is its ability to inhibit the activities of proteins such as PKC, neuraminidases, BACE1, and others (Table 2). In the case of PKC, it results in the dysregulation of signaling pathways associated with the homeostasis of the cells and cancer development (Figure 2). Even though the exact mechanisms of PKC activity inhibition by hispidin are unclear, it certainly acts as a non-competitive inhibitor and has high selectivity against PKC [19]. Hispidin also inhibits the activity of a number of other proteins which cause an overall reduction of both oxidative stress and inflammation (Figure 2).

In addition, hispidin affects the levels of pro- and anti-apoptotic proteins from the Bcl-2 family (Table 2). Hispidin may manipulate their ratio that results in the controlled death of cancer cells and the survival/protection of healthy cells. However, the exact mechanisms of hispidin action on the regulation of apoptosis in different cells still remain unknown and need to be elucidated.

Hispidin is often studied as a fungal extract component in combination with other substances. Thus, the first report about the anti-allergic effect of hispidin for rat basophilic leukemia-2H3 (RBL2H3) cells was published several years ago [106]. It was demonstrated that the anti-allergic characteristics of fungal extracts become stronger with hispidin presence. These investigations reveal new properties of hispidin that should be studied more comprehensively.

## Figures and Tables

**Table 1:** Semilethal hispidin dose (IC_50_) for various cancer cell lines.

Cell Line	Approximate Semilethal Hispidin Dose, mol/L
Skin squamous cell carcinoma SCL-1 [19]	1 × 10^−4^
Pancreatic ductal adenocarcinoma Capan-1 [19]	Between 1 × 10^−4^ and 1 × 10^−3^
Rectal carcinoma CMT-93 [28]	7 ± 1 × 10^−4^
Colorectal carcinoma HCT 116 [28]	7 ± 1 × 10^−4^
Lung carcinoma A549 [29]	2.5 × 10^−4^
Endocervical adenocarcinoma SGC-7901 [30]	6.1 ± 1.1 × 10^−3^
Pancreatic ductal adenocarcinoma BxPC-3 [31]	1 × 10^−4^
Pancreatic ductal adenocarcinoma AsPC-1 [31]	2 × 10^−4^

**Table 2:** Biological activities of hispidin mentioned in the review. Abbreviations of the proteins: BACE1—β-site amyloid precursor protein cleaving enzyme 1, GPDH—glycerol-3-phosphate dehydrogenase, NF-kB—Nuclear factor-kappa B, PAK1—Serine/threonine-protein kinase 1, p66^shc^—isoform of SHC1 adaptor protein, PTP1β—protein tyrosine phosphatase 1β.

Bioactive Property of Hispidin	Cytotoxic Effect	The Effect on Carbohydrate and Lipid Metabolism	Possible Neuroprotective Effect	Possible Cardioprotective Effect	Antiviral Effect
Free radical scavenger	Direct antioxidant activity and reduction of oxidative stress				
	↓PAK1 and NF-kB signaling—anti-inflammatory activity				
			↑Gene expression of antioxidant enzymes that (but not only) control apoptosis		
Inhibitor of activities of proteins	↓Protein kinase C			↓Caspase-3 ↓Caspase-9	↓Neuraminidases
		↓Aldose reductase ↓GPDH ↓PTP1β	↓p66^Shc^		
			↓BACE1		
Regulator of pro- and anti-apoptotic proteins interplay	Tumor cell death	Survival of myoblasts		Survival of cardiomyoblasts	
Anti-apoptotic proteins levels	↓	↑		↑	
Pro-apoptotic proteins levels	↑	↓		↓	

## Data Availability

Data available in publicly accessible sources, which are listed in References.
